# Light‐induced modifications of the outer retinal hyperreflective layers on spectral‐domain optical coherence tomography in humans: an experimental study

**DOI:** 10.1111/aos.14723

**Published:** 2021-01-04

**Authors:** Thibaud Mathis, Vivien Vasseur, Kevin Zuber, Nicolas Arej, Olivier Loria, Laurent Kodjikian, Florian Sennlaub, Martine Mauget‐Faÿsse

**Affiliations:** ^1^ Department of Ophthalmology Croix‐Rousse University Hospital Hospices Civils de Lyon University of Medicine Lyon 1 Lyon France; ^2^ UMR‐CNRS 5510 Matéis Lyon 1 University Lyon France; ^3^ INSERM CNRS Institut de la Vision Sorbonne University Paris France; ^4^ Department of Ophthalmology Rothschild Foundation Paris France

**Keywords:** melanosome, optical coherence tomography, outer retinal layers, phagosome, retinal pigment epithelium

## Abstract

**Purpose:**

Numerous small hyperreflective dots (HRDs) can be seen within the hyporeflective layer between the ellipsoid zone (EZ) and the interdigitation zone (IZ) on C‐scan spectral‐domain optical coherence tomography (SD‐OCT) with a yet unknown variation under light conditions. The aim of this study was to explore light‐induced SD‐OCT changes in these HRDs.

**Methods:**

The study subjects were randomly assigned to two groups: Group 1 experienced a dark adaptation protocol followed by intense retinal photobleaching, while Group 2, serving as the control group, was exposed to constant ambient light without any variation. The number of HRDs was automatically counted.

**Results:**

Twenty healthy volunteers were prospectively included. The number of HRDs differed significantly over time (p = 0.0013). They decreased in Group 1 after dark adaptation and retinal photobleaching before returning to baseline levels 30 min later; conversely, they remained relatively constant in Group 2 throughout the study (p < 0.001). Light‐skinned subjects had less HRD than dark‐skinned subjects.

**Conclusion:**

We observed light‐induced modifications in the space between the EZ and the IZ. We hypothesize that the HRDs visible in this zone correspond to melanosomes that are mobilized during the light stimulation protocol. Larger studies are recommended to further evaluate and confirm light‐induced SD‐OCT changes under physiological and pathological conditions.

## Introduction

Multimodal imaging techniques are improving over time, continuously bringing new perspectives for exploring the retinal structures. Spectral‐domain optical coherence tomography (SD‐OCT) has been, in the last 10 years, the most widely used technique for this purpose. With an image resolution approaching cellular dimensions (approximately 5.6 *μ*m), most recent SD‐OCT devices open new fields of exploration that until now have been possible only through histology or adaptive optical imaging. Unlike adaptive optics, SD‐OCT is a fast examination that can be performed without pupillary dilation. B‐scans and more recently C‐scans, also known as ‘en‐face OCT’, enable the visualization of the retina in the axial and transverse views, respectively.

**Fig. 1:**
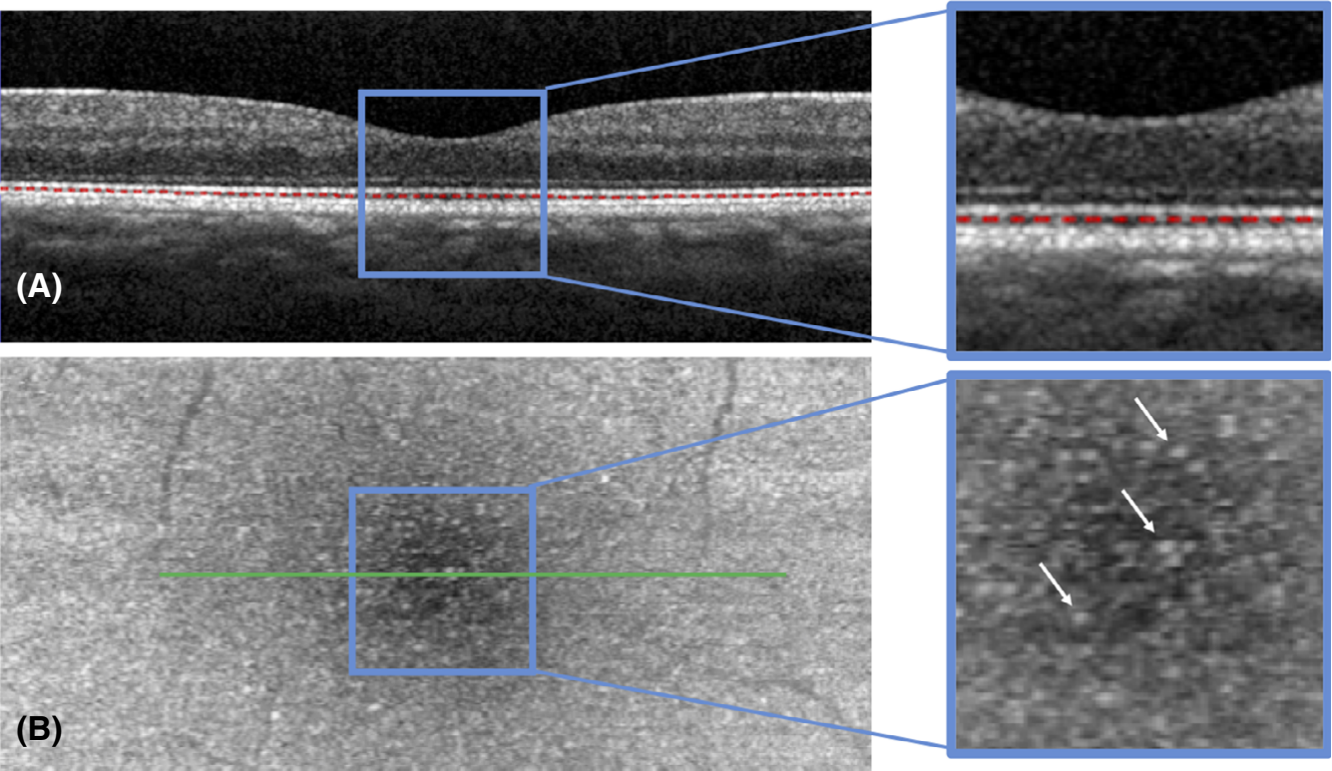
Spectral‐domain optical coherence tomography (SD‐OCT) B‐scan (A) with a red dotted line drawn between the ellipsoid and interdigitation hyperreflective zones. This segmentation was obtained by placing the line 14 *µ*m below the ellipsoid zone. The corresponding C‐scan (B) revealing numerous hyperreflective dots (arrows).

Several retinal layers have been defined according to their reflectivity on SD‐OCT and are supposed to correspond to the histological retinal layers (Staurenghi et al. 2014). This correlation between histology and reflectivity was made from SD‐OCT an interesting tool for the study of retinal structural changes (Spaide & Curcio 2011). Although many of the OCT‐defined layers are consensually assigned to specific retinal structures, the OCT–histology correlation for some of these layers remains controversial (Jonnal et al. 2014; Spaide 2015; Cuenca et al. 2018a, 2018b; Curcio et al. 2018). Recently, four hyperreflective layers were defined in the outer retina on SD‐OCT and were thought to belong to the photoreceptors or retinal pigment epithelium (RPE). It is generally accepted that they correspond to the external limiting membrane (ELM), ellipsoid zone (EZ), interdigitation zone (IZ) and the RPE layer (Staurenghi et al. 2014). Recently, Cuenca et al questioned this definition and proposed to change the terminology of the aforementioned layers. In particular, they proposed the term ‘phagosome zone’ instead of IZ for the third outer retinal hyperreflective layer based on immunohistological findings (Cuenca et al. 2018b). Several authors have studied the movement of phagosomes and melanosomes in the RPE during dark and light adaptation in fish (Hodel et al. 2006; Wilk et al. 2017) and frogs (Mondragón & Frixione 1989; Zhang et al. 2013), and demonstrated SD‐OCT changes between the EZ and the RPE under extreme light conditions. If phagosomes and/or melanosomes were responsible for the reflectivity changes, the latter would occur near the IZ which corresponds to an area between the apical side of the RPE and its apical processes. In humans, the hyporeflective space observed between the EZ and the IZ contains numerous small hyperreflective dots (HRDs) of around 10–20 *µ*m on C‐scan SD‐OCT, regularly distributed over the macular area (Fig. 1), with a yet unknown variation under light conditions.

The aim of this study was to explore light‐induced SD‐OCT changes occurring between the EZ and the IZ in human eyes.

## Subjects and Methods

### Participants

This was a prospective study, registered on ClinicalTrials.gov under the number NCT03326908. It comprised of healthy volunteers who were enrolled at the Department of Ophthalmology of Rothschild Foundation Hospital (Paris, France) from October 2018 to February 2019. This study, which adhered to the tenets of the Declaration of Helsinki, was approved by the local review board. All study subjects provided fully informed written consent before participation. Volunteers were examined before inclusion to rule out ocular or systemic diseases. Participants who were under 18 years old or who had ocular or systemic diseases were excluded from this study. Twenty healthy individuals, balanced between light‐skinned and dark‐skinned, were included. Subject characteristics such as age, gender and ethnicity were recorded in a case report form (CRF) allocated for each subject. Best‐corrected visual acuity (BCVA) was tested before examination and was reported in Snellen equivalent.

### SD‐OCT acquisition and imaging analysis

**Fig. 2:**
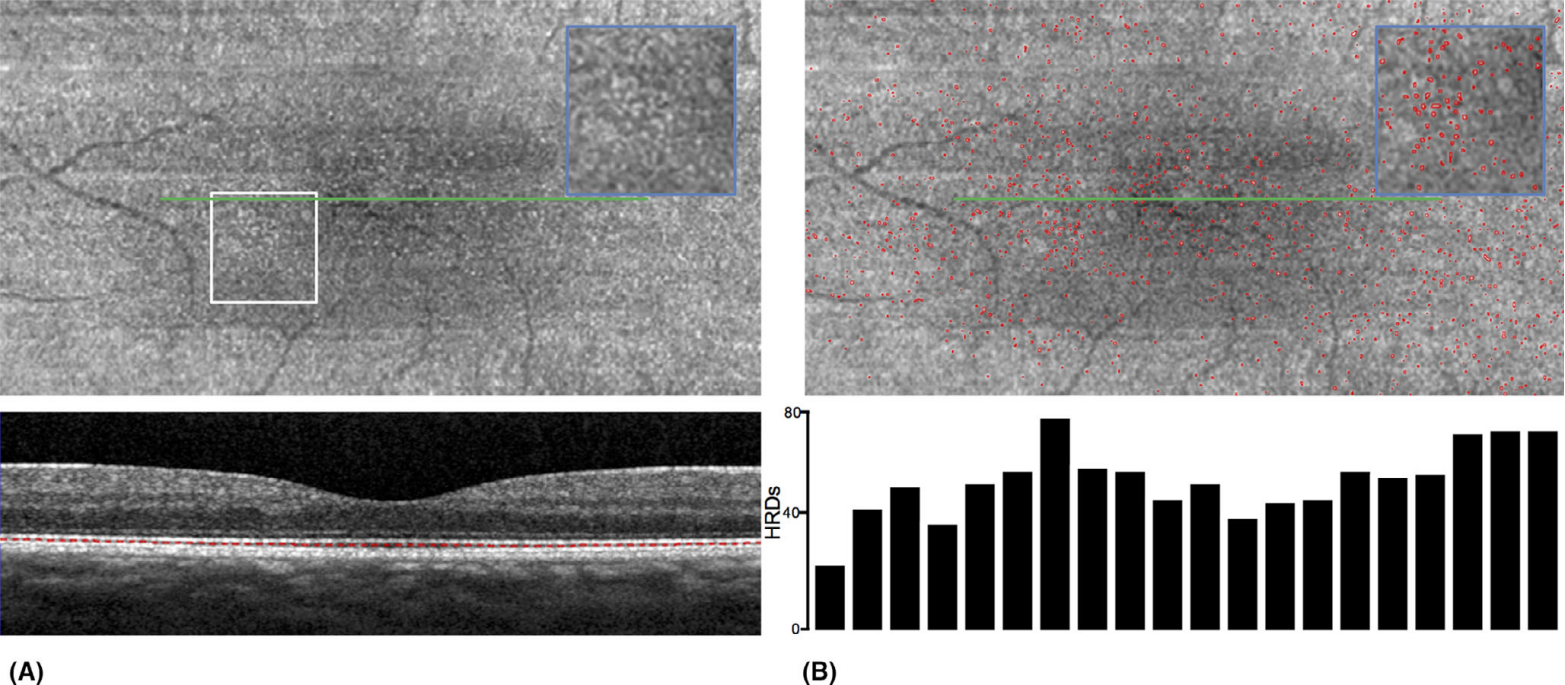
Software protocol for counting the hyperreflective dots (HRDs): before (A) and after (B) the automatic counting. Inset: close‐up view corresponding to the white box. HRDs were summed across the visual field along the *x*‐axis per 0.5 degree of the fovea and plotted their number below the panel B.

B‐scan SD‐OCT images were acquired with the SPECTRALIS HRA II – OCT device (Heidelberg Engineering, Heidelberg, Germany) without pupillary dilation. An initial retinal scan of 10° × 5° (128 slices HR, summed ART 7), carefully centred on the fovea, was performed. Quality control of the imaging was performed by two retinal specialists, and the examination was repeated until sufficient quality was obtained. During the study period, all the scans were performed in the study subjects using the follow‐up mode. The HRDs were counted on C‐scan SD‐OCT by placing the segmentation 14 *µ*m below the EZ, in an area that corresponds to the hyporeflective layer between the EZ and the IZ (Fig. 2A). After scan acquisition, images were exported from the heidelberg software (Heidelberg explorer v1.10.2.0, Heidelberg Engineering) to identify the HRD using an automated image analysis program (Aphelion v4.4.0, ADCIS, Saint‐Contest, France). A white top‐hat transform was used to extract the white dots. An opening by a disc was performed, followed by a subtraction, and then a fixed threshold. The small dots were removed by a small erosion followed by a reconstruction. The final output was a binary image with only the object of interest. Once the detection was accurate, a Voronoi tessellation was performed, based on the computation of a watershed. The distance between the dots was computed by looking at the nearest neighbour. The minimum value of the watershed is half the distance between the object and its closest neighbour.

The total number of HRDs was counted at each time‐point and recorded in the CRF (Fig. 2B).

### Imaging protocol

**Fig. 3:**
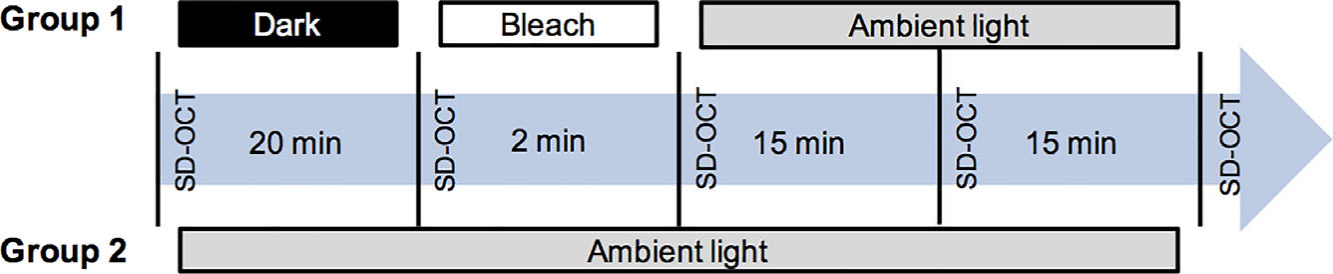
Study protocol for the two groups. Subjects in Group 1 (light stimulation protocol) experienced 20 min of dark adaptation (dark) followed by 2 min of retinal photobleaching (bleach) and then two periods of 15 min of ambient light (ambient). Group 2 (no‐light stimulation protocol) underwent optical coherence tomography imaging at each time‐point in ambient light.

The study subjects were randomly assigned to two groups: Group 1 (light stimulation protocol) and Group 2 (no‐light stimulation protocol), serving as the control group. In Group 1, after the initial SD‐OCT acquisition in ambient light (400 lux), the subjects were placed in complete darkness (0 lux) for 20 min before undergoing their second SD‐OCT acquisition. Then, they were submitted to retinal photobleaching (using the 488 nm wavelength excitation light of the autofluorescence mode on the SPECTRALIS HRA II device, 1000 lux) for 2 min, immediately followed by a third SD‐OCT acquisition. The study subjects then waited for two periods of 15 min each in ambient light (400 lux) before undergoing the fourth and fifth SD‐OCT acquisitions. In Group 2, participants underwent SD‐OCT acquisition at the same time as in Group 1, but they were kept in ambient light (400 lux) without any light stimulation (Fig. 3).

### Outcome measures

The main outcome measure was to compare the variation in the number of HRDs on C‐scan SD‐OCT between the two study groups. Other analyses included the comparison of the variation in HRD number according to skin colour.

### Statistical analysis

Numbers of HRD at baseline and at each time‐point were compared. Means and standard deviations (±SD) were reported, and the Shapiro–Wilk test was used to verify the normality of the observed distributions. A two‐way repeated‐measures anova was carried out for comparisons. All statistical analyses were performed using r version 3.4.3 (R Foundation for Statistical Computing, Vienna, Austria). For all tests, p < 0.05 was considered as statistically significant.

## Results

Twenty eyes of 20 subjects were included in this study. Ten subjects were dark‐skinned and 10 light‐skinned. There were 14 women and six men with a mean (±SD) age of 31.3 (±7.9) years; mean ages were comparable among participants with different skin colours (30.2 and 32.5 years in light‐skinned and dark‐skinned subjects, respectively). All subjects had a minimal BCVA of 1.0 Snellen equivalent. There were no significant differences between the two groups at baseline (Table 1). All subjects underwent the complete SD‐OCT acquisition protocol according to their group, and C‐scan SD‐OCT was evaluated in all cases. We first assessed the method by comparing the number of HRDs in horizontal versus vertical scans for six eyes. It appeared that the number of HRDs did not differ in an eye between horizontal and vertical scans (955.0 ± 73.1 for horizontal scan versus 962.0 ± 93.7 for vertical scans, p = 0.69 Wilcoxon's signed‐rank test). Moreover, HRD appeared to be homogeneously distributed over the macular area (Fig. 2B).

**Table 1:** Subject characteristics at baseline.

	Patient with light stimulation protocol (Group 1)	Patient with constant illumination protocol (Group 2)
Mean age, years (SD)	29.3 (±4.5)	33.4 (±10.2)
Sex ratio, % female	70%	70%
Dark‐skinned/light‐skinned ratio	1:1	1:1
BCVA, Snellen equivalent (SD)	1.1 (±0.1)	1.2 (±0.01)
Mean number of HRDs at baseline, number (SD)	898.2 (±162.9)	909.0 (±203.2)

BCVA = best‐corrected visual acuity, HRDs = hyperreflective dots.

**Fig. 4:**
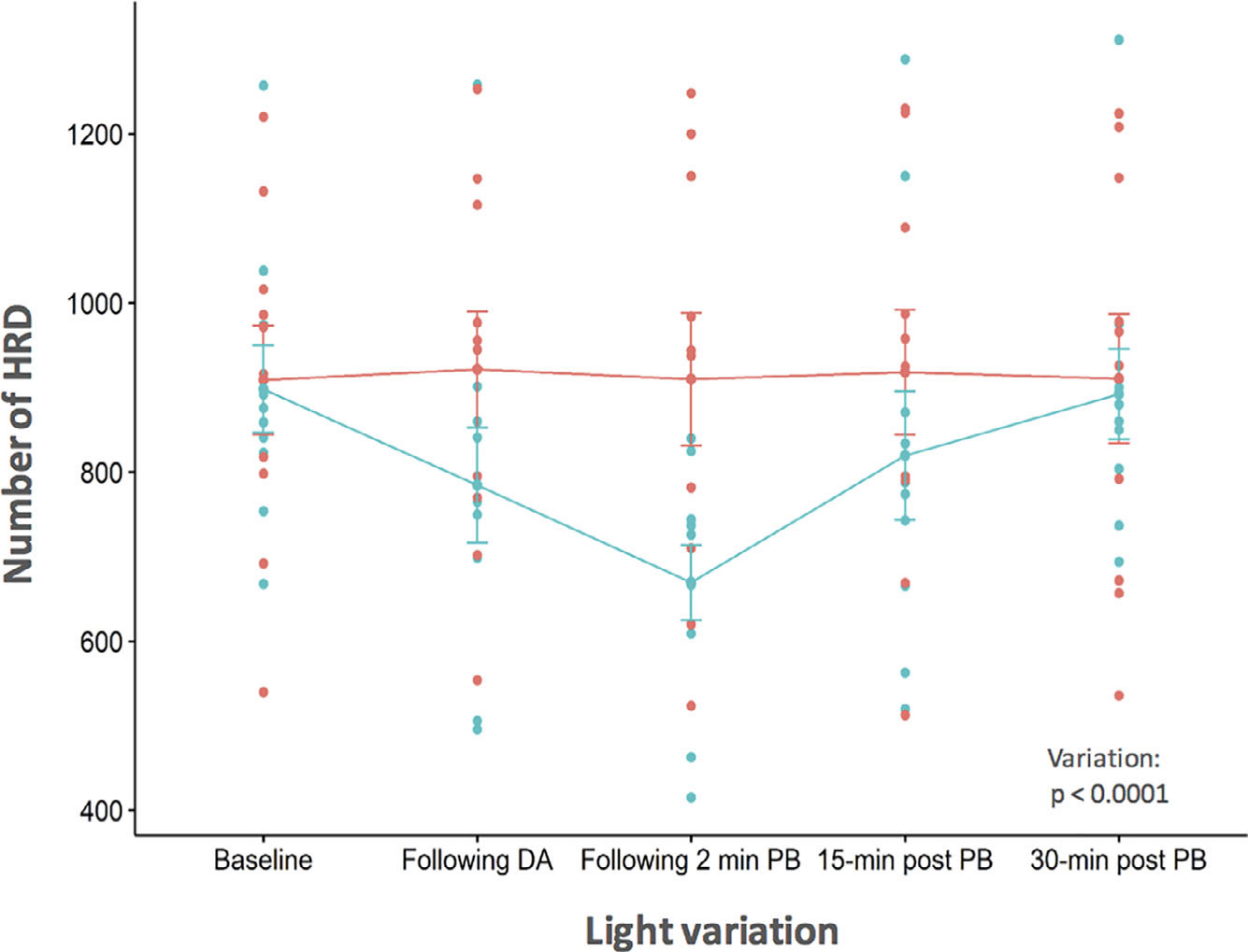
Number of hyperreflective dots (HRDs) with time between the light stimulation (blue, Group 1) and no‐light stimulation (red, Group 2) groups. Error bars represent the 95% confidence interval. DA = dark adaptation, PB = photobleaching.

For the overall population, the mean number of HRDs (±SD) differed significantly over time (one‐way anova p = 0.0013): it started high (903.6 ± 179.3) before falling after dark adaptation (853.0 ± 221.9) and further after retinal photobleaching (789.8 ± 231.7); then, it progressively returned to the baseline level by the end of the study (901.5 ± 203.3; Fig. 3). Considering the entire imaging protocol, numbers of HRD were not significantly different between the two groups (p = 0.27). As for the evolution of the number of HRDs during the protocol, they decreased after dark adaptation and retinal photobleaching before returning to baseline levels 30 min after bleaching in Group 1 (*n* = 10, group with light stimulation), while they remained very similar throughout the study in Group 2 (*n* = 10, constant illumination). The interaction effect was significant between time and light stimulation, and the main effect of time was significant (p < 0.001; Table 2 and Fig. 4). Similarly, the mean percentage change from baseline of HRD evolves in the same way during the protocol, showing a decrease in the numbers of HRDs from baseline after dark adaptation (−13.3 ± 12.8%) and after photobleaching (−25.1 ± 12.2%), in comparison with the relative stable number of HRDs from baseline in eyes with constant illumination (Table 3).

**Table 2:** Comparison of the mean number of hyperreflective dots (HRDs), at each time‐point, in the study groups.

	Baseline (*T* = 0 min)	After dark adaptation (*T* = 20 min)	After photobleaching (*T* = 22 min)	15 min after photobleaching (*T* = 37 min)	30 min after photobleaching (*T* = 52 min)
Light stimulation (Group 1)	898.2 ± 162.9	784.6 ± 215.1	669.5 ± 140.5	819.7 ± 240.3	892.2 ± 169.0
Constant illumination (Group 2)	909.0 ± 203.2	921.5 ± 217.5	910.0 ± 247.8	918.1 ± 232.1	910.7 ± 241.9

Means are presented with standard deviations.

**Table 3:** Mean percentage change from baseline of hyperreflective dots (HRDs) at each time‐points.

	Baseline (*T* = 0 min)	After dark adaptation (*T* = 20 min)	After photobleaching (*T* = 22 min)	15 min after photobleaching (*T* = 37 min)	30 min after photobleaching (*T* = 52 min)
Light stimulation (Group 1)	Ref	–13.3 ± 12.8%	−25.1 ± 12.2%	−8.5 ± 21.3%	−0.1 ± 11.1%
Constant illumination (Group 2)	Ref	0.1 ± 3.9%	0.5 ± 10.3%	−0.6 ± 8.7%	0.4 ± 8.9%

Means are presented with standard deviations.

The light‐skinned (*n* = 10) and dark‐skinned (*n* = 10) subgroups had similar behaviours over time when considering both light protocols (p = 0.97), so non‐significant interaction was seen between skin colour and time. However, there was a significant main effect of time (p = 0.002) with a sharp decrease in HRD in both subgroups after dark adaptation and after retinal photobleaching. Concerning skin colour differences in Group 1, HRD appeared to be more numerous in the dark‐skinned subgroup (*n* = 5) compared with the light‐skinned subgroup (*n* = 5), with a median (interquartile range) of 876 (859–892) versus 823 (754–974) at baseline; 770 (765–860) versus 750 (699–841) after dark adaptation; 737 (667–744) versus 669 (609–726) immediately after retinal photobleaching; 788 (774–834) versus 743 (563–871) 15 min after photobleaching; and 880 (860–910) versus 804 (737–900) 30 min after photobleaching. The median was given here due to very low number of patients in each subgroup.

**Fig. 5:**
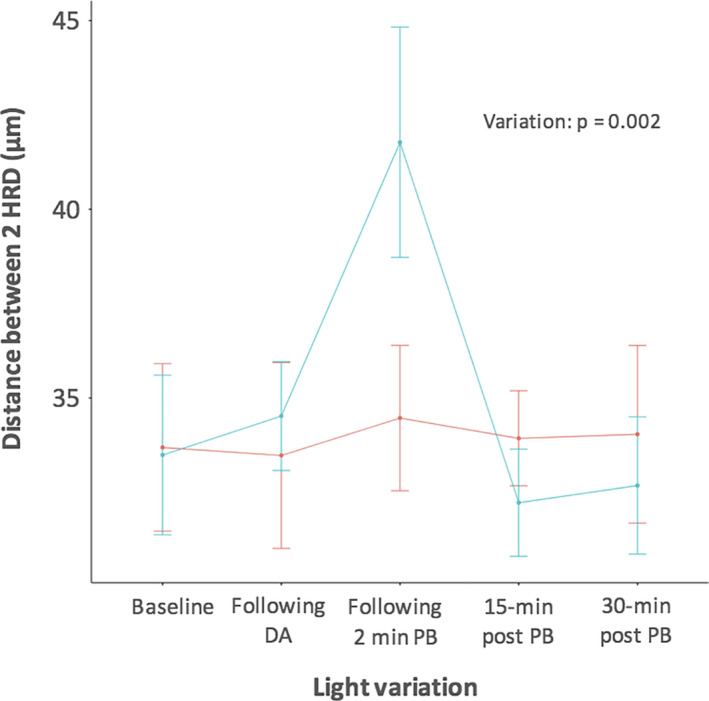
Comparison of the mean distance from a hyperreflective dot (HRD) to its nearest neighbour between the light stimulation (blue, Group 1) and no‐light stimulation (red, Group 2) groups. Error bars represent the 95% confidence interval. DA = dark adaptation, PB = photobleaching.

The distance from an HRD to its nearest neighbour was not significantly different between Groups 1 and 2, considering the entire imaging protocol (p = 0.69). Nevertheless, the distance increased in Group 1 (*n* = 10) after dark adaptation and after retinal photobleaching before returning to baseline levels 30 min after bleaching, while it remained very stable in Group 2 (*n* = 10) throughout the entire study (p = 0.002; Fig. 5).

## Discussion

This experimental study dealt with light‐induced SD‐OCT changes in the outer retinal layers. HRD seen between the EZ and the IZ significantly decreased after dark adaptation and retinal photobleaching, and then gradually increased 15 and 30 min later to reach back baseline levels. Conversely, no changes were observed in the control group that was not exposed to light stimulation protocol. We also showed that the distance between two HRDs increased after dark adaptation and retinal photobleaching compared with values at baseline and to those obtained in the control group.

There are still passionate debates about the histological correspondence of the outer retinal structures visualized on SD‐OCT, and more precisely, about the composition of the IZ (Cuenca et al. 2018a, 2018b; Curcio et al. 2018). The junction between the photoreceptors and the RPE is complex. The cone outer segment tips were previously described as ensheathed by the apical digitations of the RPE known as the contact cylinder (Spaide & Curcio 2011). Several melanosomes are present in these processes and play an active role in photoprotection, like a sunshade that goes up and down according to light stimulation with an increase in the number of melanosomes during light adaptation (Mondragón & Frixione 1989; Zhang et al. 2013).

Several authors have already shown light‐induced variations in the outer retinal hyperreflective layers in animals. Zhang et al hypothesized that melanosomes can contribute to OCT reflectivity in the RPE and showed that frog eyes exposed to light stimulation had their outer retinal layers modified. In particular, they observed that an 8‐hr ambient light adaptation resulted in an internal displacement of the more external hyperreflective outer retinal layer (Zhang et al. 2013). Berger et al have demonstrated in mice that a light challenge over 4 days led to a temporary abolition of the distinction between the two bands defining the photoreceptor outer segments and corresponding to the EZ and the IZ in human eyes (Berger et al. 2014). While RPE hyporeflectivity was seen in albinos mice, it has not been established whether human albinism could induce reflectivity changes of the RPE (Berger et al. 2014). In fact, Chong et al reported normal – even increased – reflectivity of the RPE in human albinos (Chong et al. 2009). In the present study, although the light‐induced HRD changes were statistically similar between light‐ and dark‐skinned subjects, it appears that dark‐skinned individuals tend to have more HRDs. This could be likely due to the higher number and volume of melanosomes in dark‐skinned subjects (Hurbain et al. 2018). It has already been shown in mice that light could induce changes between the RPE and photoreceptor tips within few minutes, which translate into an enlargement of the hyporeflective band between these two layers. The authors hypothesized that light adaptation induced a thickening of the outer retina due to fluid accumulation in the subretinal space (Li et al. 2016). Other authors have observed changes in outer retinal thickness during light stimulation and have attributed these modifications to photoreceptors’ physiology (Lu et al. 2017; Zhang et al. 2017).

**Fig. 6:**
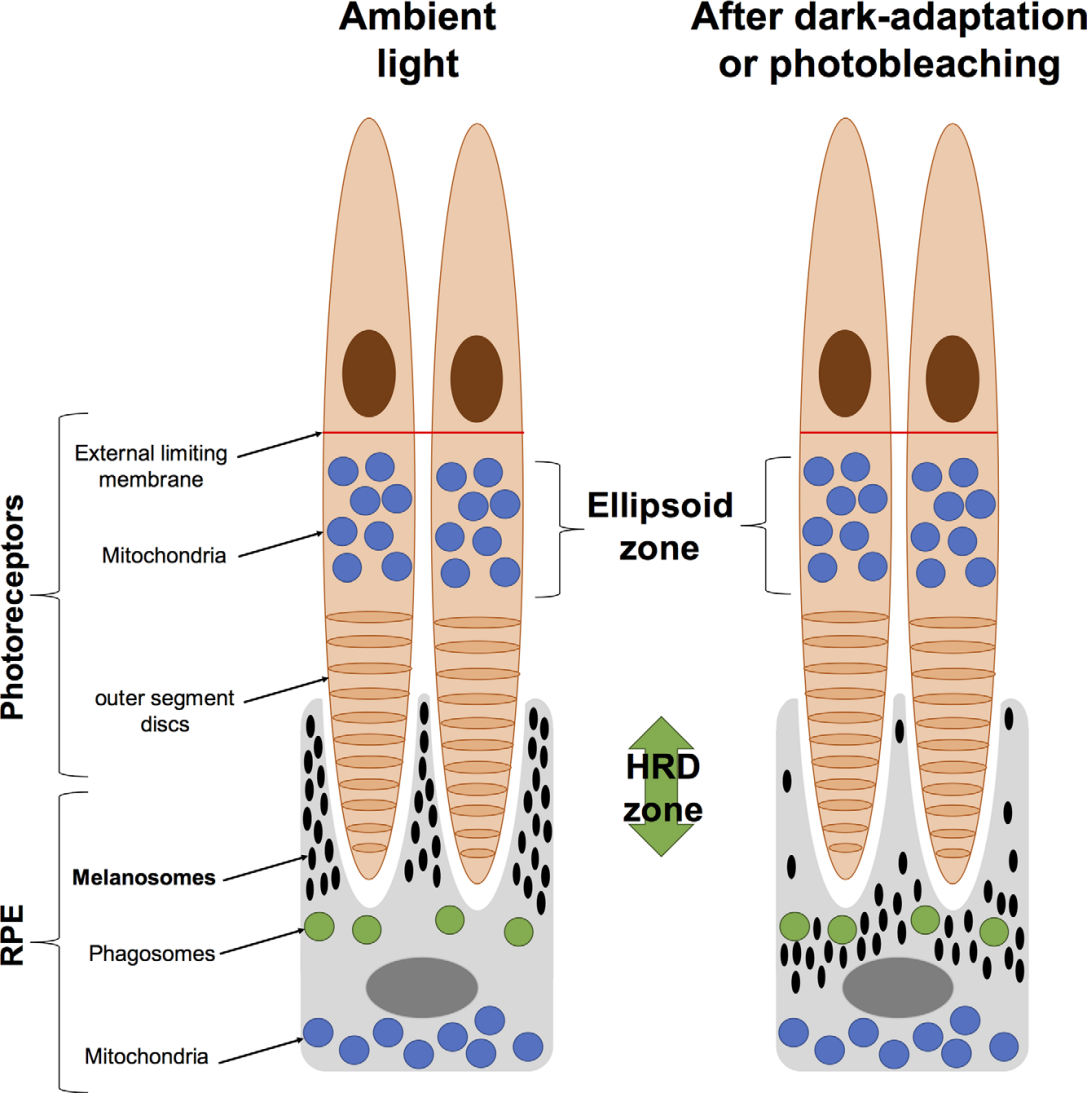
Diagram showing the hypothetic correlation between hyperreflective dots (HRDs) and melanosome movements in ambient light, dark adaptation or photobleaching. Melanosomes are recruited through the retinal pigment epithelium (RPE) apical processes to protect the photosensitive outer segments from light exposure. After dark adaptation, melanosomes are confined in the basal area of the processes.

In our study, we chose to evaluate the modifications in the space between the EZ and the IZ because it hosts the apical processes of the RPE and thus the movements of melanosomes. The latter are known to move into the apical processes of the RPE depending on light stimulation (Mondragón & Frixione 1989) and are easily imaged as melanin is one of the major contributors to the intensity of SD‐OCT hyperreflectivity (Wilk et al. 2017). In ambient light conditions, melanosomes move into the apical processes of the RPE to protect the photosensitive photoreceptor outer segments. After dark adaptation, fewer melanosomes are present as the need to protect retinal photosensitive structures decreases. These physiological modifications are consistent with our findings regarding the evolution of HRD numbers over time, and hence, we assume that the presence of HRD represents the movement of melanosomes in the RPE apical processes (Fig. 6). Besides, organelles translocate in the cells by cytoskeletal scaffolds or microfilaments/microtubules and it has been shown that the proteins constituting the scaffolds are subject to modification under irradiation by blue light. Light‐induced oxidative stress results in cytoskeletal disruption, slowing the translocation of organelles such as melanosomes and phagosomes (Burke & Zareba 2009). In our study, retinal photobleaching using a 488 nm blue light for 2 min may have induced such an oxidative stress (Teussink et al. 2017), resulting in a decreased number of melanosomes in the apical processes of the RPE that explains the significant decrease in HRD. This decline was even more pronounced after photobleaching compared with the dark adaptation period that preceded it.

Advances in retinal imaging and improvements in OCT technology have enhanced the definition of retinal structures and helped to better understand the correspondence between histology and imaging. Moreover, the study of changes in retinal structure during physiological conditions such as light stimulation can help unravel and monitor the biological mechanisms that occur during the visual process.

With respect to study limitations, the small number of included subjects may be considered as a weakness. Therefore, future large‐scale studies are recommended to confirm our findings. Also, one could consider that the HRDs seen on C‐scan SD‐OCT are artefacts (speckle noise), but the high intra‐individual reproducibility among subjects within the control group compared with the changes seen in subjects who experienced light stimulation protocol are arguments against this line of thought. Furthermore, we have counted the same numbers of HRDs in both horizontal and vertical scans, which also contradicts speckle noise assumptions. The study protocol was designed to show the variation of HRD under multiple light conditions. As such, we chose to start the bleaching on dark‐adapted eyes where translocation of organelles is already low (Zhang et al. 2013) to further lower it by light challenge (photobleaching) in order to show a higher difference from baseline. In the same way, we have planned to return to baseline adaptation level after photobleaching to compare the group with light stimulation to the group with no light stimulation at the end of the protocol. Other protocols could have also been tested in different settings. Finally, exact comparisons between histology and SD‐OCT images can be challenging as retinal thickness and other pathology features may be altered after death and sample preparation. Consequently, definitive labelling of retinal layers on SD‐OCT is quite difficult and outer retinal structures with specific reflectivity are referred to as ‘zones’ (Staurenghi et al. 2014). Novel emerging OCT technologies such as photothermal OCT and polarization‐sensitive OCT are interesting tools that could possibly be used to optimize the study of the outer retina (Cense et al. 2009; Lapierre‐Landry et al. 2018a, 2018b).

## Conclusion

Changes to the space between the EZ and the IZ can occur in human eyes in response to light stimulation. The HRD observed on SD‐OCT imaging of this zone could possibly correspond to melanosomes that are mobilized (extended) during light adaptation. Dark adaptation caused a significant decrease in HRD numbers, and bleaching after dark adaptation caused a further decrease. Further studies are needed to more thoroughly assess light‐induced changes under physiological and pathological conditions.
